# Comprehensive Candidate Gene for Ginsenoside Rg1 Biosynthesis: Identification, Systematic Analysis, and Verification

**DOI:** 10.3390/plants15131987

**Published:** 2026-06-26

**Authors:** Ruicen Liu, Dinghui Wang, Ge Jin, Li Li, Chaofan Wang, Yanfang Wang, Kangyu Wang, Mingzhu Zhao, Yi Wang, Meiping Zhang

**Affiliations:** 1 College of Life Science, Jilin Agricultural University, Changchun 130118, China; liuruicen777@163.com (R.L.);; 2 College of Chinese Medicinal Materials, Jilin Agricultural University, Changchun 130118, China; 3 Jilin Engineering Research Center Ginseng Genetic Resources Development and Utilization, Jilin Agricultural University, Changchun 130118, China

**Keywords:** association analysis, Rg1 biosynthesis, gene interaction, functional validation, *Panax ginseng*, SNP/InDel correlation

## Abstract

Ginsenoside Rg1 has a broad spectrum of pharmacological activities, and its concentration is a key indicator of ginseng quality. Because ginsenoside levels are quantitative traits controlled by multiple genes and environmental factors, identifying genes involved in Rg1 biosynthesis is essential to improve its production in ginseng. Transcriptome analysis of 344 4-year-old ginseng roots identified 33 Candidate Genes implicated in Rg1 biosynthesis. These candidates were detected through differential expression analysis, SNP/InDel mutation screening, correlation analysis with Rg1 content variation, and co-expression network analysis. By integrating GO functional annotation, gene expression profiles, and Rg1 content correlations, a putative biosynthetic pathway for Rg1 was inferred and the roles of these genes were preliminarily clarified. Further study of MeJA regulation confirmed that *PgRg1-021* is highly associated with ginsenoside Rg1 biosynthesis. Functional verification was performed via overexpression of *PgRg1-021* and RNAi in ginseng hairy roots. RT-qPCR analysis showed that *PgRg1-021* negatively regulated ginsenoside Rg1 biosynthesis: Rg1 saponin levels decreased after overexpression and increased after RNAi. This study was the first to verify *PgRg1-021*’s role in Rg1 biosynthesis and provided essential gene resources for pathway analysis. It established a foundation for targeted breeding and serves as a reference for identifying other genes associated with complex traits.

## 1. Introduction

*Panax ginseng* C.A. Meyer is a perennial herb in the family *Araliaceae* and genus Ginseng. It is both a valuable traditional Chinese medicine with high therapeutic value and a health-care product used to support daily well-being [1,2,3,4,5,6]. Ginsenosides are the primary active compounds responsible for ginseng’s pharmacological effects [6], and their activities differ markedly because of structural and physicochemical variation. Among them, ginsenoside Rg1 is designated in the Pharmacopeia of the People’s Republic of China as a key marker of ginseng quality due to its high abundance and broad pharmacological profile. To achieve a comprehensive understanding of ginsenoside biosynthesis, the critical task is to screen for and identify the functional genes that govern this pathway [7,8]. The biosynthetic pathway of Rg1 has been clarified, particularly the key enzyme genes that catalyze its formation [9,10,11,12,13]. In one route, glycosylation at PPT C-20 yields F1, a reaction catalyzed by UGTPg71A53 and UGTPg71A55; subsequent glycosylation at the C-6 position of F1, catalyzed by UGTPg71A54 and UGTPg71A55, produces Rg1. In the alternative route, UGTPg71A54 first glycosylates PPT at C-6 to form Rh1 [9,13,14,15,16,17]. In addition, UGTPg71A29 can also convert ginsenoside Rh1 into ginsenoside Rg1 in yeast [9,13,14].

The catalytic biosynthesis pathway of Rg1 provides a solid foundation for further comprehensive and in-depth research on Rg1 biosynthesis. However, ginsenoside biosynthesis is a complex, multi-step biological process that should not be restricted to studies of catalytic activity alone. As quantitative traits, ginsenosides are controlled by many genes with small, similar effects that can act cumulatively and interact with environmental factors. Consequently, an in-depth, comprehensive screen of the gene clusters involved in ginsenoside Rg1 biosynthesis is required. Current methods for identifying key functional genes underlying biological traits primarily include quantitative trait locus (QTL) mapping [18,19,20,21] and genome-wide association studies (GWAS) [21,22]. The ginseng genome is approximately 3.6 Gb in size [23] and highly repetitive [24]. These genomic features, together with ginseng’s long reproductive cycle, have limited development of high-quality genetic maps and artificial mapping populations, which in turn constrain the effective application of QTL and GWAS approaches for identifying functional genes in ginseng.

To address these challenges, some researchers have analyzed ginseng transcriptomic data using single-association or correlation methods, including differentially expressed gene (DEG) analysis [25], gene expression [17,26,27], single-nucleotide polymorphism (SNP) mutation [28,29], and gene co-expression interactions [30]. However, results from these single-correlation approaches were too broad to pinpoint specific target genes; consequently, they were mainly useful for validating the roles of known ginsenoside biosynthetic enzymes rather than for discovering novel functional genes.

Building on the preceding research, this study systematically integrated those methods to screen DEGs between two ginseng sample groups that differed markedly in Rg1 content. Key Candidate Genes involved in ginsenoside Rg1 biosynthesis were identified through multiple analytical approaches, and the function of *PgRg1-021*, which has the highest correlation with ginsenoside Rg1 content, was verified. *PgRg1-021* as an AAA-ATPase gene, its role in saponin biosynthesis had not been previously reported. The study discovered several novel genes associated with ginsenoside biosynthesis and provided a practical framework for screening genes tied to complex traits. It also delivered critical insights into the molecular mechanisms regulating ginsenoside Rg1 biosynthesis and supplied genetic resources for synthetic biology and targeted production of ginsenoside Rg1.

## 2. Materials and Methods

### 2.1. Databases and Materials

Database I (NCBI/GEO SRR13131364–SRR13131405 and SRR23758499–SRR23758802) represents the transcriptome of 4-year-old ginseng roots and comprises 344 samples. It contains nucleotide sequences for 248,993 transcripts, TPM expression values for all transcripts, SNP and insertion/deletion (InDel) mutations, and ginsenoside content measured for the samples (including Rg1) [31].

Database II is the transcriptome of MeJA-elicited ginseng adventitious roots sampled at multiple treatment durations. It was assembled using the above transcriptome as the reference and therefore contains the expression profiles of those transcripts under MeJA treatment. The database also includes the mono-ginsenoside content corresponding to each sample, including Rg1 concentrations [32].

The vectors and organisms used in the experiment were stored in the laboratory.

### 2.2. Identification of DEGs Involved in Rg1 Biosynthesis

Using the Rg1 measurements from 344 samples in the 4-year-old ginseng root transcriptome database I, samples were ranked in descending order and examined with box plots. After removing outliers identified in the box plots, a continuous population of samples based on Rg1 content was established for further analysis. From this population, the 30 samples with the highest Rg1 content (high group) and the 30 samples with the lowest Rg1 content (low group) were selected for differential expression analysis because of their marked phenotypic contrast. DEG analysis was performed with the DESeq2 package in R Studio (Version 1.3.1093) using a false discovery rate threshold of 0.1 (*p*-adj ≤ 5.0 × 10^−2^). In the screening of genes regulating ginsenoside biosynthesis, to ensure thatCandidate Geneses with subtle but functionally significant changes in expression were not overlooked, we employed two methods for selection: First, we performed a preliminary screening using a more lenient threshold (FDR = 0.1) to enhance sensitivity. Subsequently, we applied a stricter criterion (*p*-adj ≤ 5.0 × 10^−2^) to the results to further screen the significance of each DEG, ensuring these genes possess higher statistical significance and reducing the risk of false positives. DEG were designated as Candidate Gene involved in Rg1 biosynthesis and were renamed “*PgRg1-number*”. Principal component analysis (PCA), volcano plot, and expression heatmap of these DEGs were generated with TBtools software (Version 1.047).

### 2.3. Analysis of the Relationship Between Candidate Gene I Expression and Rg1 Content

Pearson correlation analysis was performed via SPSS (IBM SPSS Statistics 23) between TPM expression of Candidate Gene I in 344 samples and the corresponding ginsenoside Rg1 content. Genes showing a significant correlation (*p*-value ≤ 5.0 × 10^−2^) with changes in ginsenoside Rg1 content were designated Candidate Gene II.

### 2.4. Analysis of SNP/InDel Mutations Significantly Associated with Changes in Rg1 Content in Candidate Gene II

The SNP and InDel mutations of the Candidate Gene II were extracted from Database I. Samples were then grouped by the genotype at each SNP or InDel site, and SPSS software was used to calculate the significance of differences in Rg1 content between groups. Mutation sites whose variation significantly correlated with Rg1 content, along with their corresponding genes, were designated Candidate Gene III. 

### 2.5. Construction of Co-Expression Network for Candidate Gene III

Based on the TPM expression of Candidate Gene III in the 4-year-old ginseng root transcriptome (Database I), a co-expression network was constructed to identify its significant interactions. Network analysis was performed in R Studio (Version 1.3.1093) and visualized with BioLayout Express3D (Version 3.3). Genes that form a co-expression network with key enzyme genes are considered closely related to those enzymes and may participate in ginsenoside biosynthesis. Therefore, the expression levels of 15 key enzyme genes (*SS_1*, *SE2_1*, *SE2_4*, *DS_1*, *DS_3*, *FPS_22*, *CAS_11*, *CAS_22*, *AS_1*, *AS_6*, *CYP716A52v2_3*, *CYP716A53v2_1*, *CYP716A47_1*, *UGT71A27_2*, and *UGT74AE2*) were incorporated to construct the network, resulting in the identification of Candidate Gene IV, which displayed significant interactions. Multiple co-expression networks were established for all genes at *p*-value thresholds ranging from 5.0 × 10^−2^ to 1.0 × 10^−8^, repeating the process 20 times. More than half of the genes, including Candidate Gene IV and 15 key enzyme genes, were included in these networks. Concurrently, an equal number of genes were randomly selected from the transcriptome to build negative-control networks. Subsequent statistical analysis of node and edge quantities across different *p*-value thresholds confirmed that the network’s compactness was not a random occurrence.

### 2.6. Analysis of GO Functions and Enrichment for Candidate Gene IV

To investigate the functions of Candidate Gene IV, the present study performed Gene Ontology (GO) annotation using Blast2GO software (Version 5.2.5) and conducted enrichment analysis with the OmicShare web tool (www.omicshare.com), using all DEGs as the control. GO annotations in the categories of biological process (BP), molecular function (MF), and cellular component (CC) were determined and analyzed statistically.

### 2.7. Analysis of the Relationship Between Candidate Gene IV and Rg1 Biosynthesis Key Enzyme Genes

Genes with complete Open Reading Frames (ORF) (ORFfinder Home—NCBI) and Conserved Domains (CD-Search: New Query) were identified via NCBI database retrieval. Based on known gene functions, the relationships among these genes, key enzymes in ginsenoside biosynthesis, and ginsenoside Rg1 content were investigated. Correlation coefficients for gene expression levels and the associations between gene expression and Rg1 content were calculated using SPSS. Candidate Gene IV for the Rg1 pathway was then ranked by expression correlation coefficient from highest to lowest and mapped into the pathway with arrows directed toward Rg1.

### 2.8. MeJA Regulation Analysis of Candidate Gene IV

Methyl jasmonate (MeJA) significantly increases ginsenoside content, including Rg1, in ginseng adventitious roots [12], so it serves as a tool to test genes implicated in ginsenoside biosynthesis. Expression data of Candidate Gene IV involved in Rg1 biosynthesis were extracted from the transcriptome of MeJA-elicited ginseng adventitious roots (Database II), and their expression levels were compared with the control group using SPSS. Genes whose expression changed significantly after MeJA treatment were taken as responsive and may play important roles in ginsenoside biosynthesis.

### 2.9. Functional Validation of the Target Gene Involved in the Biosynthesis of Ginsenoside Rg1

Primer Premier 5.0 was used to design the forward and reverse primers for target gene, and the gene was amplified from cDNA. The amplified fragment was ligated into the pMD™ 18-T vector and transformed into *E. coli* DH5α competent cells; the constructs were sent to the company for sequencing. After a 100% sequence match was confirmed, the pCAMBIA3301 overexpression vector and the RNA interference (RNAi) vector were constructed. Single-digest analysis verified successful construction of the pCAMBIA3301 overexpression vector. The RNAi system used binary vectors assembled from amplified forward and reverse interfering fragments generated with gene-specific primers. Sense and antisense fragments were cloned into a cloning vector and then transferred into the intermediate vector pHANNIBAL. The RNAi-containing fragment was excised and ligated into pART27, and double-digest analysis confirmed the successful construction of the pART27 RNAi vector.

The recombinant gene expression vector was transformed into competent cells of engineered *Agrobacterium* C58C1 to obtain positive engineered strains. The bacterial suspension was used to infect young adventitious ginseng roots cultured on B5 solid medium for about 30 days at 22°C. First, the young adventitious roots were pre-cultured on MS solid medium at 22 °C for 48 h to achieve an optimal state. Second, the roots were cut into 2–3 mm long segments, infected with the activated suspension of genetically engineered positive strains, and cultured for 24 h. Third, the segments were transferred to 1/2 MS solid medium containing antibiotics for selection and growth, and their growth was observed and recorded regularly until maturation.

### 2.10. To Detect the Change in Rg1 Synthesis Gene Expression and Saponin Content in Transgenic Hairy Roots

Three wild-type controls, three pCAMBIA3301 overexpression-positive hairy roots asexual lines, and three pART27 RNAi-positive hairy roots asexual lines were cultured for a period of time. From each root system, 1 g of tender tissue was collected and cultured for 30 days, with three replicates per group. RNA was extracted and reverse transcribed into cDNA, which served as the template for RT-qPCR performed on an ABI 7500 Fast Real-Time PCR System. Perform three technical replicates during PCR sample loading to minimize loading errors. Because the target gene is closely related to ginsenoside Rg1 biosynthesis, three key enzyme genes in the Rg1 synthesis pathway (*SE2-1*, *CYP716A47*, and *UGT101*) were selected for RT-qPCR. The reference gene was a *CYP* gene [12], and primers for RT-qPCR were designed with Primer Premier 5.0. The experiment included three technical replicates and three biological replicates. Relative gene expression levels were calculated by the 2^−ΔΔCt^ method with normalization to the reference gene. After confirming the change in gene expression, single transgenic positive hairy-root samples were dried and ginsenosides were extracted by Soxhlet extraction. Ginsenoside content was determined by high-performance liquid chromatography (HPLC). Chromatographic and mobile-phase elution conditions were adopted from the relevant literature [12,33]. Take the average of three replicates of each single root system. RT-qPCR and HPLC analysis statistical test methods are both *t*-test.

## 3. Results

### 3.1. Screening of DEGs in Ginsenoside Rg1 Biosynthesis

**Figure 1:**
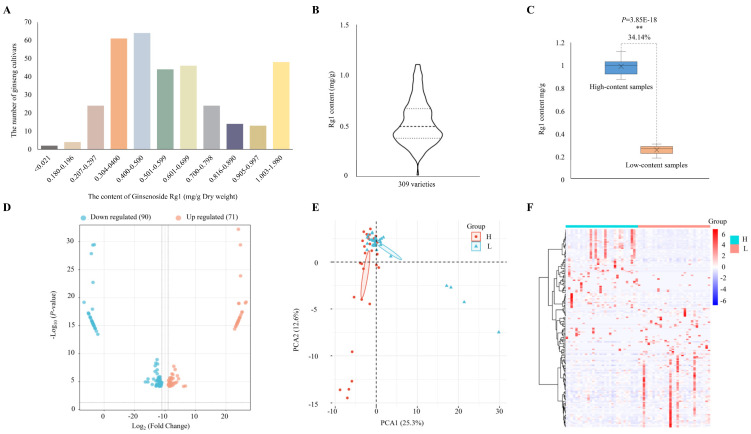
Rg1 content distribution in the subpopulation and DEGs. (**A**) Distribution of Rg1 contents in 344 ginseng samples. (**B**) Rg1 contents in the subpopulation excluding Rg1 content outliers. (**C**) Rg1 contents in the high- and low-content groups. ** indicates a statistically significant difference at *p* < 0.01. (**D**) Volcano map of 161 DEGs with 90 down-regulated and 71 up-regulated. (**E**,**F**) PCA and Heatmap of 161 DEGs expressions in the H- and L-groups.

All 344 transcriptome samples from 4-year-old ginseng roots were ordered by Rg1 content. The distribution of sample counts across content ranges followed a normal distribution (Figure 1A). To ensure reliable downstream analysis, 35 outliers were removed, yielding 309 samples with continuous Rg1 content (Figure 1B). In this set, the median Rg1 content was 0.542 mg/g, the upper quartile 0.678 mg/g, and the lower quartile 0.384 mg/g. The narrower contour above the median and the broader contour below it indicated that Rg1 content was concentrated around the median and lower quartile. From these samples, the 30 highest and 30 lowest Rg1 content samples were subsequently selected (Figure 1C). The mean Rg1 content in the high group was 0.990 mg/g and in the low group 0.252 mg/g, a roughly threefold difference (*p*-value = 3.85 × 10^−18^).

DEG analysis between the two groups was conducted using the DESeq2 package in R Studio. From the transcriptome, 161 significant DEGs were identified at *p*-adj ≤ 5.0 × 10^−2^, of which 71 were up-regulated and 90 were down-regulated (Figure 1D). The PCA plot of these DEGs showed completely separated confidence ellipses for the high and low groups, indicating clear differences between them (Figure 1E). The heatmap likewise revealed markedly different expression patterns between the high and low groups (Figure 1F). Accordingly, these 161 DEGs were designated Candidate Gene I (Supplementary Table S1).

### 3.2. Correlation Analysis Between the Expression of Candidate Gene I and Rg1 Content

The expression profiles of the 161 DEGs were extracted from 344 transcriptome samples of 4-year-old ginseng, and Pearson correlation analysis was performed between their expression levels and the corresponding Rg1 contents. 65 DEGs showed a significant correlation with Rg1 content (*p*-value ≤ 5.0 × 10^−2^) and were designated Candidate Gene II (Supplementary Table S2).

### 3.3. SNP/InDel Mutation Analysis of Significant Correlation Between Candidate Gene II and Rg1 Content Change

**Figure 2:**
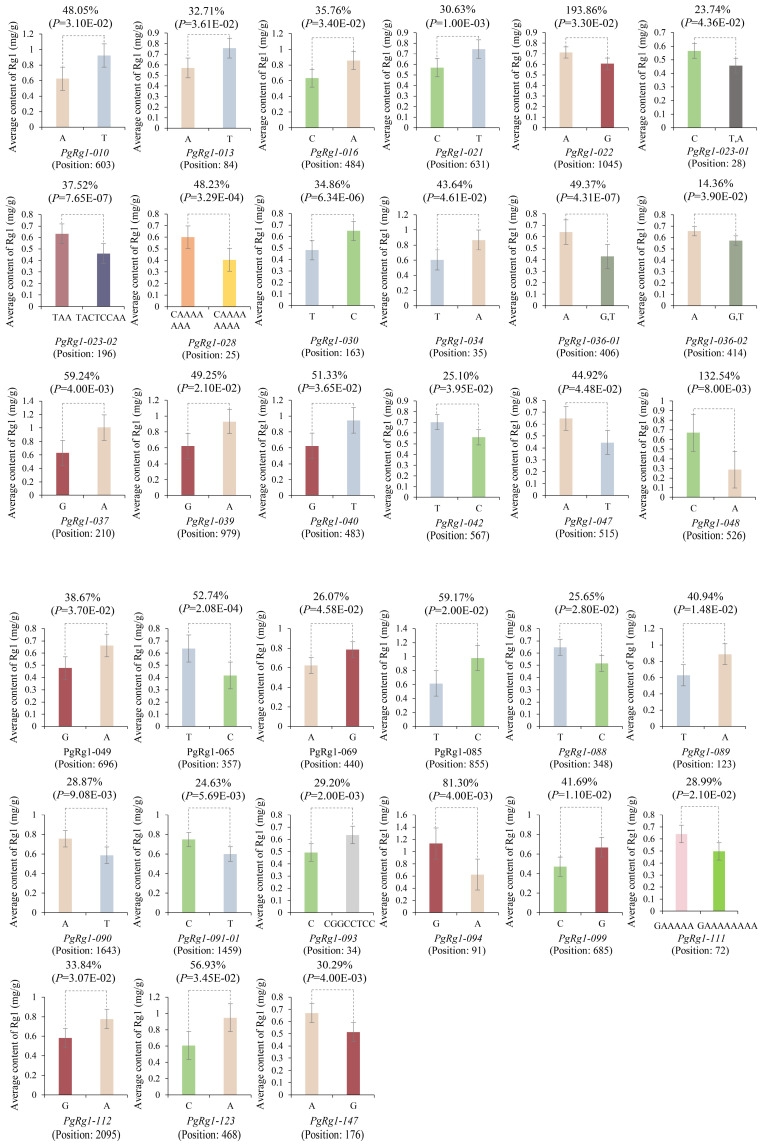
Impact of SNP/InDel mutations in the 33 Candidate Gene III on Rg1 contents. The *X*-axis shows the reference genotype (left) and SNP/InDel mutation (right) at a specific position of a Candidate Gene. The *Y*-axis was the average of Rg1 contents. The impact degree of each mutation on the average Rg1 content was the percentage difference between two homozygous groups at a specific *p*-value.

In the mutation database of the 344 4-year-old ginseng root transcriptomes, 2274 SNP/InDel were identified across 65 genes in Candidate Gene II. These variants were used to classify the 344 samples by genotype, and SPSS was used to test for significant differences in Rg1 content among the genotype groups. The analysis showed that 141 SNP/InDel in 33 genes were significantly associated with variation in ginsenoside Rg1 content (*p*-value ≤ 5.0 × 10^−2^); these 33 genes comprised Candidate Gene III. The statistical analysis shows that the 141 SNP/InDel loci differ markedly in their effects on ginsenoside Rg1 content: the smallest effect of a single locus is 14.17% and the largest is 193.86%. Selecting, for each gene, the SNP/InDel locus with the greatest effect on Rg1 content revealed that Rg1 levels changed significantly after mutation (Figure 2, Supplementary Table S3). After mutation, Rg1 content increased significantly at 17 loci and decreased at 16 loci, which may relate to their roles in Rg1 biosynthesis.

### 3.4. Co-Expression Network Analysis of Candidate Gene III

**Figure 3:**
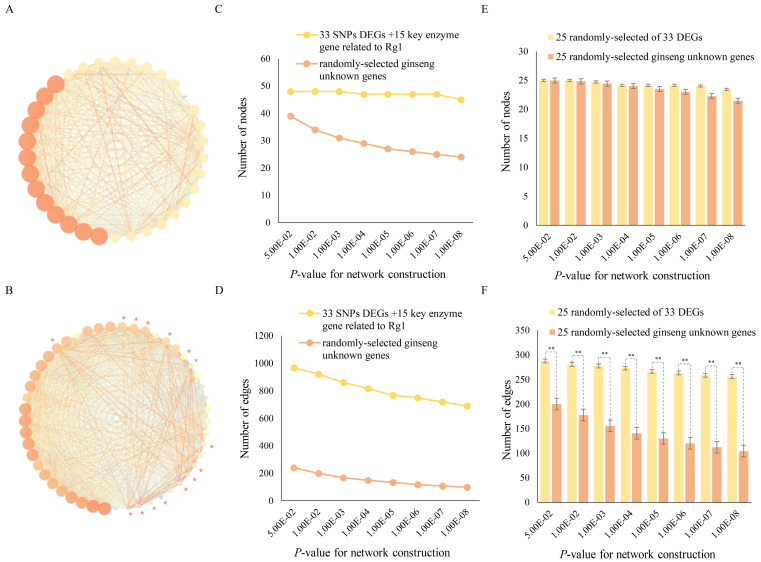
Gene co-expression networks. (**A**) Co-expression network of 33 Candidate Gene III in 344 samples. (**B**) Co-expression network of 33 Candidate Gene III and 15 key enzyme genes in 344 samples. Colored dots represent candidate genes, five-pointed stars represent key enzyme genes, and the size of the circles indicates the strength of co-expression relationships (or node connectivity weight, depending on what your figure actually shows). (**C**,**D**) Number of nodes and edges in the networks from 33 Candidate Gene III and 15 key enzyme genes, and the control group in 344 varieties at different *p*-values. The control group is randomly selected from the reference transcriptome. (**E**,**F**) Statistics of nodes and edges in the networks from 25 genes randomly selected from 33 Candidate Gene III and 15 key enzyme genes, with the control group, and repeated 20 times. ** indicates a statistically significant difference at *p* < 0.01.

To investigate the relationship between gene expression and Candidate Gene III, co-expression network analysis was performed. At *p*-value ≤ 5.0 × 10^−2^, these 33 genes formed a co-expression network with an Average Connectivity of 31.82, indicating strong co-expression and functional correlation among the genes (Figure 3A). The 33 genes also formed a co-expression network (*p*-value ≤ 5.0 × 10^−2^) with 15 key enzyme genes (marked with orange pentagrams) (Figure 3B). These results indicated that the 33 Candidate Gene III members likely participate substantially in ginsenoside biosynthesis.

To validate this finding, networks constructed from an equal number of genes randomly sampled from the transcriptome were used as the control. Comparing the numbers of nodes and edges across different *p*-values showed that the gene co-expression network composed of Candidate Gene III and key enzyme genes exceeded the control network (Figure 3C,D). This was further statistically validated by constructing networks from 25 randomly selected genes per group, with the sampling process repeated 20 times. Counting nodes and edges at different *p*-values again showed that networks of Candidate Gene III plus key enzyme genes had higher node and edge counts than the control (Figure 3E,F).

This finding further confirms the accuracy of Candidate Gene identification by examining gene interactions and indicates that the significant co-expression between Candidate Gene III and key enzymes likely reflects a functional mechanism rather than random association. Overall, the 33 Candidate Gene III identified in this study were considered to be involved in ginsenoside Rg1 biosynthesis.

### 3.5. GO Function Annotation and Enrichment Analysis of Candidate Gene IV

**Figure 4:**
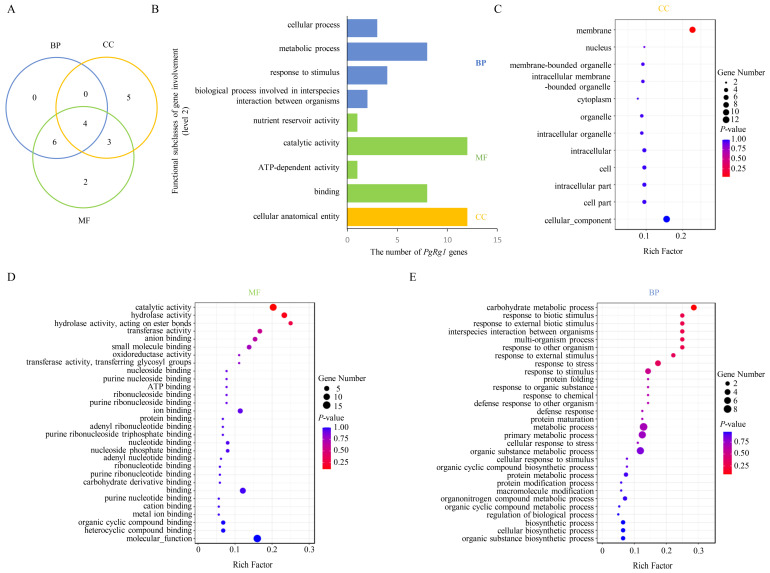
GO annotation and enrichment analysis of 33 Candidate Gene III. (**A**) Venn diagram of the three functional categories. (**B**) Functional subcategories (Level 2). (**C**) CC, (**D**) MF, (**E**) BP enrichment analysis. BP: biological process, MF: molecular function, and CC: cellular component.

Using GO functional annotation and enrichment analysis, 20 genes were classified into three primary categories: 10 genes in BP, 12 in CC, and 15 in MF (Figure 4A). These categories were further divided into nine secondary subcategories (Figure 4B). The metabolic process, catalytic activity, binding, and cellular anatomical entity subcategories contained the largest numbers of Candidate Genes. GO enrichment analysis was performed for 33 Candidate Gene III using all 161 DEG GO annotations as the background. The most enriched subcategories were membrane in CC (Figure 4C), catalytic activity in MF (Figure 4D), and carbohydrate metabolic process in BP (Figure 4E).

### 3.6. The Relationship Among Candidate Gene IV and the Rg1 Biosynthesis Key Enzyme Genes in the Pathway

**Figure 5:**
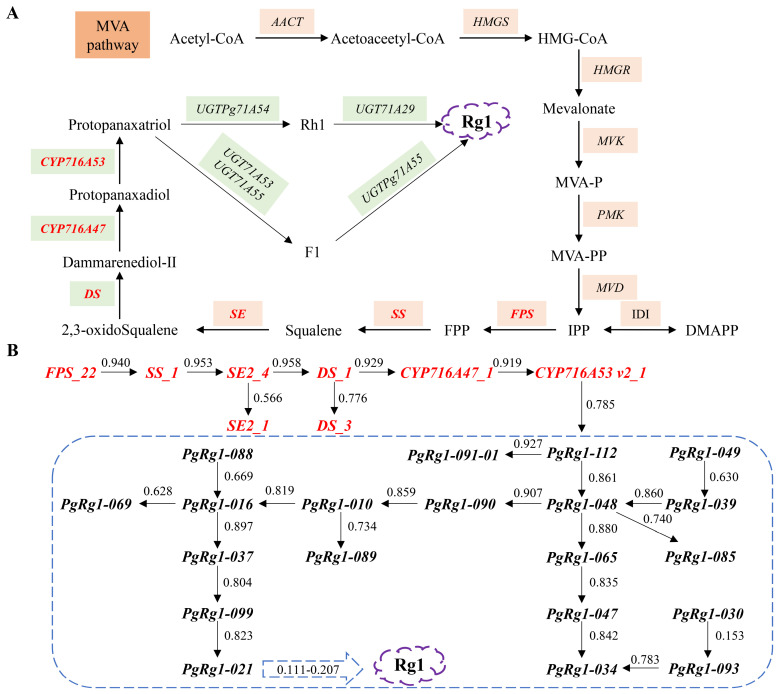
Pathway of the 8 key enzyme genes, 20 Candidate Genes, and Rg1. (**A**) Biosynthesis pathway of ginsenoside Rg1. (**B**) Pathway of 20 Candidate Genes related to ginsenoside Rg1 synthesis and 8 key enzyme genes involved in ginsenoside Rg1 synthesis. The arrow points to phenotype Rg1, and its value represents the correlation coefficient between genes.

To assess the roles of the 20 functionally annotatedCandidate Geneses in the ginsenoside Rg1 metabolic pathway, key enzyme genes were selected from the Rg1 biosynthetic pathway (Figure 5A), and the putative positions of each Candidate Gene within the pathway were inferred based on their correlation coefficients with Rg1 content (Figure 5B). The key enzyme genes (red font) clustered together and exhibited highly significant correlations (*p*-value ≤ 1.00 × 10^−2^). Pairwise correlation coefficients among these genes ranged from 0.628 to 0.907 (*p*-values ≤ 5.0 × 10^−2^–1.00 × 10^−8^). Correlation coefficients between the 20 Candidate Gene and Rg1 content ranged from 0.118 to 0.207 (*p*-values ≤ 5.0 × 10^−2^–1.00 × 10^−3^). These results indicate that genes with related functions localize to the same region of the pathway, which indicates that they are highly correlated in gene expression.

### 3.7. Expression Analysis of Candidate Genes Regulated by MeJA

**Figure 6:**
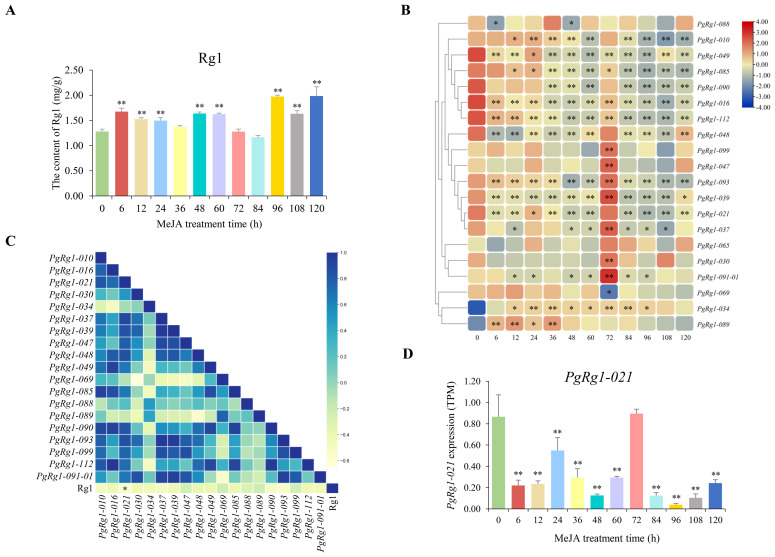
MeJA treatment for different time points. (**A**) Variation in Rg1 contents in the ginseng adventitious roots treated with MeJA for different time points. (**B**) Expression heatmap of the 20 Candidate Genes in response to MeJA treatment for different treatment time points. (**C**) Heatmap showing the correlation between 19 MeJA-regulated genes and Rg1 saponin content. (**D**) Changes in relative expression of the *PgRg1-021* with increasing MeJA treatment time. (* for a two-tailed significance of *p* < 0.05, ** for a two-tailed significance of *p* < 0.01).

MeJA induction significantly increases ginsenoside content, so genes that respond strongly to MeJA likely participate in ginsenoside biosynthesis. To test this, Rg1 content and Candidate Gene expression in MeJA-treated ginseng adventitious roots were compared with those in the control (0 h) using the MeJA-induced transcriptome of ginseng adventitious roots (Database II). Relative to the control (0 h), Rg1 content increased significantly at all measured time points except 36 h, 72 h, and 84 h (*p*-values ≤ 5.00 × 10^−2^ or 1.00 × 10^−3^), confirming that MeJA regulated Rg1 biosynthesis (Figure 6A). Likewise, the expression of 19 of the 20 Candidate Genes changed significantly at one or more time points after MeJA treatment (Figure 6B), indicating these genes responded positively to MeJA and thereby affected ginsenoside biosynthesis (Supplementary Table S4). Thus, the functions of 19 Candidate Genes in Rg1 biosynthesis were preliminarily verified. Among these 19 genes (Figure 6C), only *PgRg1-021* showed a significant correlation with ginsenoside Rg1 content (*p*-value ≤ 5.00 × 10^−2^) (Supplementary Table S5). After MeJA treatment, *PgRg1-021* expression changed significantly at all time points except 72 h (Figure 6D), indicating a close association with Rg1 biosynthesis. Consequently, *PgRg1-021* was selected as the target gene for subsequent functional verification.

### 3.8. Cloning and Vector Construction of the PgRg1-021

The full-length ORF of *PgRg1-021* is 1464 bp. Specific primers were designed (F: 5′-ATGTTTTCTTTGAATAGTGTGCCTT-3′; R: 5′-TTAGAATCCTACCTTCTGTTTCTTGC-3′). The ORF forward and reverse primers for *PgRg1-021* included *Xma* I restriction site and protective bases. The overexpression vector was ligated to produce pCAMBIA3301-*PgRg1-021* and was validated by single digestion with *Xma* I. The 201 bp fragment of *PgRg1-021* was cloned in both sense and antisense orientations to construct the RNAi vector pART27-*PgRg1-021*, which was confirmed by single digestion with *Not* I (Supplementary Figure S1). Sequencing was performed after each cloning step, and the sequencing results matched the original sequence 100%.

### 3.9. Genetic Transformation of Ginseng Adventitious Roots and Detection of Positive Hairy Roots

**Figure 7:**
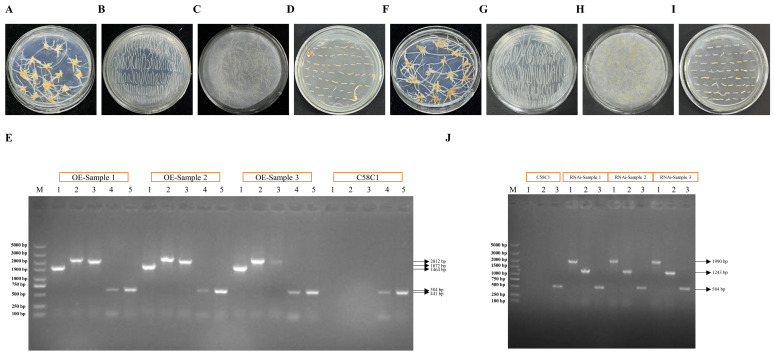
Infection process of adventitious roots of recombinant vector ginseng and hairy roots asexual line positive test. (**A**,**F**) Adventitious root culture. (**B**,**G**) Pre-culture. (**C**,**H**) Co-culture. (**D**,**I**) Hairy root screening culture. (**E**) Positive test of overexpression vector pCAMBIA3301-*RgRg1-021* hairy roots. M: DL5000; 1: *RgRg1-021*; 2: upstream portion of vector sequence containing *RgRg1-021*; 3: downstream portion of vector sequence containing *RgRg1-021*; 4: *GFP* gene; 5: *rol* C gene (**J**) Positive test of RNAi vector pART27-*RgRg1-021* hairy roots. M: DL5000; 1: upstream portion of vector sequence containing *RgRg1-021*; 2: downstream portion of vector sequence containing *RgRg1-021*; 3: *rol* C gene.

The constructed pCAMBIA3301-*PgRg1-021* and pART27-*PgRg1-021* plasmids were introduced into *Agrobacterium* C58C1 by *Agrobacterium*-mediated transformation and then used to infect ginseng adventitious roots. Healthy ginseng adventitious roots cultivated for about 30 days (Figure 7A,F) were pre-cultured for 48 h (Figure 7B,G), then infected with bacterial suspension and co-cultured for 24 h (Figure 7C,H). After co-culture, the adventitious roots were transferred to selection medium and incubated until growth was observed (Figure 7D,I). Hairy roots induced by *Agrobacterium* exhibited hormone autotrophy and rapid growth. Ultimately, three independent hairy-root lines positive for pCAMBIA3301-*PgRg1-021* (Figure 7E) and three independent hairy-root lines positive for pART27-*PgRg1-021* (Figure 7J) were obtained, and their transgene expression levels and ginsenoside Rg1 contents were determined.

### 3.10. Relative Expression Levels of the Rg1 Synthesis Gene in Transgenic Positive Hairy Roots

**Figure 8:**
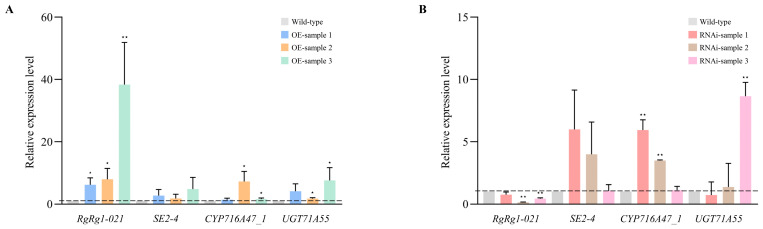
The relative expression level of the *PgRg1-021* and three key enzyme genes (*SE2-4*, *CYP7146A47_1*, and *UGT101*) in positive hairy root lines of ginseng (* for a two-tailed significance of *p* < 0.05, ** for a two-tailed significance of *p* < 0.01). The dotted line is the wild type, and the relative expression level is 1. (**A**) OE-sample 1, OE-sample 2, OE-sample 3 genetically transformed with pCAMBIA3301-*PgRg1-021.* (**B**) RNAi-sample 1, RNAi-sample 2, RNAi-sample 3 were genetically transformed with pART27-*PgRg1-021.*

The three pCAMBIA3301-*PgRg1-021* overexpression hairy-root lines showed significantly increased *PgRg1-021* expression to varying degrees (Figure 8A). OE-sample 3 exhibited the largest increase, with a relative expression 38.32 times that of the wild type, demonstrating successful overexpression of *PgRg1-021* in ginseng hairy roots. Conversely, the three pART27-*PgRg1-021* RNAi hairy-root lines displayed significant decreases in *PgRg1-021* expression (Figure 8B). RNAi-sample 2 showed the greatest change, with a relative expression 14.4-fold lower than the wild type, confirming successful RNAi of *PgRg1-021* in ginseng hairy roots. The three known Rg1 biosynthetic catalytic genes were differentially upregulated following both overexpression and RNAi treatments, with their relative expression levels markedly higher after RNAi, which may reflect negative regulation of Rg1 biosynthesis by *PgRg1-021*.

### 3.11. Changes in Saponin Content in Transgenic Hairy Roots

**Figure 9:**
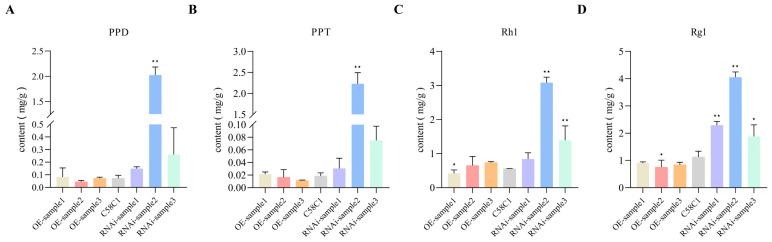
The (**A**) PPD, (**B**) PPT, (**C**) Rh1, (**D**) Rg1 saponin contents of OE-sample 1, OE-sample 2, OE-sample 3 genetically transformed with pCAMBIA3301-*PgRg1-021*, BK C58C1, and RNAi-sample 1, RNAi-sample 2, RNAi-sample 3 genetically transformed with pART27-*PgRg1-021* (* for a two-tailed significance of *p* < 0.05, ** for a two-tailed significance of *p* < 0.01).

HPLC was used to quantify four ginsenosides—PPD, PPT, Rh1, and Rg1—with wild-type C58C1 ginseng hairy roots as controls (Supplementary Figure S2). Changes in each ginsenoside in genetically transformed hairy roots were compared with those in the controls. Figure 9 shows that Rg1 content decreased in all positive hairy roots after overexpression of *PgRg1-021*. Conversely, RNAi of *PgRg1-021* significantly increased Rg1 content in positive hairy roots. Notably, Rg1 in OE-sample 2 positive hairy roots fell to 0.75 mg/g, a 33.6% reduction relative to the control. In contrast, Rg1 in RNAi-sample 2 positive hairy roots rose to 4.056 mg/g, a 259.26% increase versus the control. These results further confirm that changes in Rg1 content after genetic transformation are consistent with a negative regulatory role of *PgRg1-021* on Rg1. Moreover, PPT and Rh1 showed similar trends along the PPD-to-Rg1 synthesis pathway.

## 4. Discussion

Ginsenoside metabolism is a central topic in ginseng research because it reflects both the plant’s growth characteristics and its medicinal value. Nevertheless, progress in this area has lagged behind that for other secondary metabolites such as artemisinin [34,35]. To date, most key enzyme genes that catalyze ginsenoside biosynthesis [13,15,36] and some regulatory factors [37,38] have been identified and validated. However, comprehensive pathway of ginsenoside biosynthesis still needs more information, including additional key enzyme genes, transcriptional regulators, and genes required to supply substrates and energy. Addressing these gaps requires a study with broad coverage, high accuracy, and high efficiency. Therefore, this study investigated Candidate Genes involved in ginsenoside Rg1 biosynthesis to expand current knowledge.

Two sample sets with markedly different Rg1 contents were first selected from a population of 344 ginseng samples to ensure the accuracy of subsequent analyses. Based on the Rg1-content phenotype differences, Differential gene expression analysis was conducted with an initial threshold of FDR = 0.1; FDR values were further adjusted by *p*-value (*p*-adj ≤ 5.0 × 10^−2^) to control the false-positive rate. Subsequently, correlation analyses between Rg1 content and DEG, association analyses between Rg1 content and gene SNP/InDel genotypes, and gene co-expression correlation analyses were performed sequentially, each using a *p*-value ≤ 5.0 × 10^−2^. Single correlation tests can produce results even due to random chance, which can affect research accuracy; however, the analysis platform used in this study addressed this issue. By integrating correlations and associations across gene expression, gene mutation, and phenotype, Results with a combined *p*-value below 5.0 × 10^−6^ were obtained. Therefore, the 33 Candidate Genes identified here were the genes most strongly associated with Rg1 biosynthesis in ginseng, suggesting their key roles in that pathway.

*PgRg1-021*, as an AAA-ATPase, provides energy through ATP hydrolysis and participates in DNA replication, protein degradation, peroxisome formation, membrane fusion, signal transduction and gene expression regulation [39,40,41,42]. This study found that this gene can influence the content of ginsenoside Rg1. *PgRg1-021* overexpression in this research lowered Rg1 accumulation, whereas RNAi interference increased Rg1 accumulation, demonstrating that *PgRg1-021* negatively modulates Rg1 production and accumulation. At the same time, *SE2-1*, *CYP716A47*, and *UGT101* were elevated in *PgRg1-021* overexpression and RNAi. However, the rise in transcriptional levels does not necessarily cause a commensurate increase in protein levels, enzyme activity or metabolic flux, nor necessarily correlate with an increase in the final metabolite content [43]. The accumulation of Rg1 is affected by the transcriptional levels of one or a few genes related to biosynthesis and also by multiple processes such as precursor supply, substrate competition between different ginsenoside synthesis pathways, glycosylation modification, transport and storage [15,44,45]. SE catalyzes the conversion of squalene to 2,3-oxidosqualene, which is a key rate-limiting step in the upstream pathway of triterpenoid saponin biosynthesis [44]. CYP716A47 catalyzes the conversion of Dammarenediol-II to PPD and participates in oxidative modification following the formation of the ginsenoside skeleton [45]. UGT71A55 is essential for glycosylation modification of PPT-type ginsenosides [15], and catalyzes the production of F1 from PPT, and then produces Rg1 from F1. Transcriptional overexpression of these genes does not always result in an increased net metabolic flux towards Rg1. This behavior could be associated with the alterations of metabolic flux distribution in the ginsenoside biosynthesis network.

The overexpression of *PgRg1-021* may change the precursor distribution, substrate competition, downstream glycosylation or conversion process, as well as transport and storage that may reduce the net flow towards Rg1 accumulation. In contrast, *PgRg1-021* RNAi might decrease the negative regulatory role on Rg1 accumulation and hence lead to Rg1 accumulation. Previous investigations showed that the spatiotemporal expression patterns of Rg1-related Candidate Genes were different from those of known critical ginsenoside enzyme genes, and that alterations in the gene interaction network might influence Rg1 biosynthesis [46]. Therefore, the present data reveal that *PgRg1-021* may regulate the accumulation of Rg1 via a complicated regulatory network, instead of just stimulating the transcription of many known biosynthesis-related genes. However, the precise regulatory mechanism of *PgRg1-021* on Rg1 accumulation has to be defined. The verification of this work was mainly based on the detection of transcriptional level and Rg1 content. Future investigations should combine protein level verification, enzyme activity analysis and complete metabolomics to establish if *PgRg1-021* influences the accumulation of Rg1 by altering enzyme activity, substrate distribution or downstream conversion pathways of ginsenosides.

The levels of Rg1 and its metabolites PPT and Rh1 were altered in a consistent manner after *PgRg1-021* overexpression and RNAi. This suggests that the regulatory role of *PgRg1-021* could not be restricted to the last phase of Rg1 biosynthesis but might also affect upstream events in the synthetic branching of PPT-type ginsenosides. Thus, the variations in PPT, Rh1 and Rg1 indicate that *PgRg1-021* may be involved in precursor supply, PPT production or metabolic flux partitioning in the PPT-type branch, rather than only a regulator of the last stage of Rg1 synthesis. However, we have not measured upstream common precursors and other monomeric saponins because of the constraints of experimental materials and assay conditions in this investigation. Therefore, the specific regulatory locations of *PgRg1-021* in the ginsenoside biosynthesis network are still ambiguous. In future work, we will aim to comprehensively identify the upstream precursors and ginsenoside products for the many branching pathways. This will be paired with transcriptome analysis and functional validation to more precisely identify the regulatory nodes of *PgRg1-021.*

Jasmonic acid and its derivatives regulate plant growth and development and play a role in stress resistance [17,47]. Exogenous MeJA significantly induces the accumulation of secondary metabolites, including ginsenosides [48,49,50,51]. Several studies reported that ginsenoside Rg1 content increased markedly following MeJA elicitation [12,52,53,54], and MeJA induction has been shown to up-regulate genes involved in ginsenoside biosynthesis, such as *FPS*, *SS*, *SE*, *DS*, *PPDS*, and *PPTS* et al. [55,56]. Consequently, differential transcriptome analysis under MeJA treatment has become an effective approach for initially identifying key genes in ginsenoside biosynthesis [57,58]. MeJA-regulated ginsenoside biosynthesis is dynamic and time-dependent, and does not rise continuously at all time points. This is because the metabolic response oscillates in phases, involving feedback regulation of JA signals, temporal delays between changes in gene expression and accumulation of metabolites, and dynamic balance among ginsenoside production, transformation, and transport [51,59,60]. Accumulation of a single ginsenoside component is induced in a non-continuous and non-linear manner. It is regulated by sequential expression of genes associated with biosynthesis, feedback control of jasmonic acid signals and the dynamic balance of ginsenoside synthesis, transformation and transport [59,60]. In the study, there was no significant increase in Rg1 concentration at 36, 72, and 84 h after MeJA therapy. This result does not exclude the regulatory role of MeJA in Rg1 biosynthesis. This may indicate that Rg1 is accumulated in a non-linear, time-specific manner after MeJA activation, and is regulated by both transcriptional and metabolic control.

The identified Candidate Genes are nonetheless encouraging: they represent different mechanisms of ginsenoside biosynthesis and show significant interrelationships in pathway analysis. This finding indicates that the platform can not only detect Candidate Genes but also predict their functional roles and interaction models. In follow-up studies, experiments will be designed to verify interactions among these candidates, thereby refining and validating the platform’s accuracy and reliability.

## 5. Conclusions

In this study, a variety of bioinformatics methods were integrated, including DEG analysis, SNP/InDel analysis and co-expression network analysis, and Candidate Genes highly related to ginsenoside Rg1 biosynthesis were identified. Through the correlation between gene expression patterns and Rg1 content, the potential pathways involved in Rg1 biosynthesis were preliminarily inferred, and the role of these genes in Rg1 biosynthesis was revealed. Subsequently, *PgRg1-021*, which is highly associated with ginsenoside Rg1 biosynthesis, was confirmed by MeJA acid induction. Experiments such as over-expression and RNAi show that *PgRg1-021* has a negative regulatory effect on ginsenoside Rg1 content, and its expression level is significantly related to ginsenoside Rg1 content. These results indicate that *PgRg1-021* plays a key role in the regulation of ginsenoside Rg1 biosynthesis. As an AAA-ATPase, its role in Rg1 metabolism is likely to be indirect, rather than achieved through direct catalysis of the Rg1 biosynthetic pathway. Further research is required to determine whether *PgRg1-021* interacts with specific biosynthetic enzymes, transcription factors, or other regulatory proteins involved in ginsenoside biosynthesis, and to clarify the relevant regulation mechanism of ginsenoside Rg1 biosynthesis. This will lay a theoretical foundation for analyzing the biosynthesis and regulation mechanism of ginsenoside Rg1, and provide genetic resources for ginsenoside production of homologous or heterologous species. At the same time, the platform used in this study can be used as an example for the identification and analysis of Candidate Genes for important economic traits of ginseng and other plants.

## Data Availability

The data used for this study are available at the Sequence Read Archive (SRA) of the National Center for Biotechnology Information (NCBI), BioProject PRJNA302556, and at Gene Expression Omnibus (GEO) of NCBI, SRP066368 and SRR13131364-SRR13131405.

## Supplementary Materials

The following supporting information can be downloaded at: https://www.mdpi.com/article/10.3390/plants15131987/s1, Supplementary Table S1: The differentially expressed gene transcripts were obtained by differential analysis among samples with significant differences in Rg1 content. Supplementary Table S2: The significant correlation analysis results between DEG transcripts and Rg1 content. Supplementary Table S3: The SNP/InDel mutation analysis results significantly related to the change in ginsenoside Rg1 content and the impacts of their genic SNP/InDel on Rg1 contents. Supplementary Table S4: The expression variation in Candidate Genes in the adventitious roots treated with MeJA for 6 h through 120 h, relative to the control roots. Supplementary Table S5: The significant correlation analysis results between Candidate Gene expression and ginsenoside Rg1 content in response to MeJA regulation. Supplementary Figure S1: Cloning and vector construction of *PgRg1-021*. (A) Cloning of *PgRg1-021*. M: DL2000; 1: blank; 2: target gene. (B) Construction of the expression vector pCAMBIA3301-*PgRg1-021*. (C) Enzymatic verification of recombinant plasmid pCAMBIA3301-*PgRg1-021*. M: DL15000; 1: recombinant plasmid; 2: *Xma* I single-digest validation. (D) Cloning of the sense and antisense segments of *PgRg1-021*. M: DL500; 1: blank; 2-3: sense and antisense segments of *PgRg1-021*. (E) Construction of the expression vector pART27-*PgRg1-021*. (F) Single-digest validation of recombinant interference vector pART27-*PgRg1-021*. M: DL500; 1: recombinant plasmid; 2: *Not* I single-digestion. Supplementary Figure S2: The original HPLC figures of PPD, PPT, Rh1, and Rg1 monomer saponins, over-expression, RNAi, and CK (C58C1). The *X*-axis represents time (min), and the *Y*-axis represents mAU (milli absorbance units).

## Abbreviations

BP	biological process
CC	cellular component
DEG	differentially expressed genes
FDR	false discovery rate
GO	gene ontology
GWAS	genome-wide association study
HPLC	high-performance liquid chromatography
MeJA	methyl jasmonate
MF	molecular function
NS	non-synonymous mutations
ORF	open reading frame
PPD	Protopanaxadiol
PPT	protopanaxatriol
QTL	quantitative trait locus
S	synonymous mutation
SNP/InDel	single nucleotide polymorphism and nucleotide insertion/deletion
